# Exploring Spirituality as a Forgotten Variable in the Contexts of Communities of Faith and Education

**DOI:** 10.1093/geroni/igaa057.1994

**Published:** 2020-12-16

**Authors:** Lydia Manning

**Affiliations:** Concordia University Chicago, River Forest, Illinois, United States

## Abstract

The purpose of this investigation was to describe the lived experience of aging in communities of faith, more specifically how older adults experienced social support. Semi-structured interviews were conducted with 15 older adults ranging in age from 63 to 84 years of age. Grounded Theory coding techniques were applied to identify themes. Findings include people feeling supported by community, connections, and beliefs, but also failed regarding opportunities for elder ministry or other types of worship. Participants expressed wanting more intentional opportunities for engagement. Many participants described the desire for an “expert” in their communities of faith. We consider the culture of support and how communities of faith may need to reconsider traditional ways of meeting the needs of people as they age. Furthermore, we explore the creation of educational tools that may allow for the emergence of an aging specialist in communities of faith.

